# Editorial: Healthy eating and parenting messages to prevent obesity

**DOI:** 10.3389/fpubh.2023.1177742

**Published:** 2023-04-11

**Authors:** Lisa Bailey-Davis, Jennifer S. Savage

**Affiliations:** ^1^Population Health Sciences, Geisinger Health System, Danville, PA, United States; ^2^Nutritional Sciences, The Pennsylvania State University (PSU), University Park, PA, United States

**Keywords:** dietary guidelines, parent feeding, obesity prevention, socioeconomic status, policy

National food and dietary guidelines translate science for public and professional audiences. Upstream, guidelines are established by scientific committees with the intent of informing the communications or messages of clinicians, educators, and marketers to parents, children, patients, and consumers downstream. Guidelines have historically addressed what and how much to eat, but messages about where, when, why, and how to eat or feed children have emerged in the current national guidelines. For example, the *Dietary Guidelines for Americans, 2020–2025* includes guidance for parents and caregivers of newborns that addresses developmental readiness for introducing solid foods, strategies for introducing complementary foods and beverages, and guidance for responsive feeding to support healthy eating patterns (1). Recognition that eating is influenced by habits, culture, and context is warranted to better support behavior change for health promotion and disease prevention.

This Research Topic brought together research from multiple countries about messages that address where, when, why, and how to eat or feed children with the intent of supporting healthy eating and preventing obesity. Ramuscak et al. observe improved parental awareness, knowledge, and opinions about the 2019 Canada's Food Guide compared to the earlier 2007 version. Use of the Food Guide by parents of young children remained persistently low from 2007 to 2019, but given the high recall of the plate model recommendation, the authors call for research that investigates whether practicing this recommendation translates to dietary changes. Also from North America, Shamah-Levy et al. evaluate data from six National Health and Nutrition Surveys in Mexico and report the obeso-protective effects of increased fruit and vegetable consumption among school-age children. They discuss cross-cutting environmental policies to promote the availability and consumption of sustainable and affordable foods to protect against overweight and obesity in children. Policy strategies aimed at reducing sugar-sweetened beverages remain an important topic in North America. In a qualitative study, Haynes-Maslow et al. observe that adolescents appreciate the long-term drawbacks of sugar-sweetened beverages but hold positive perceptions about consuming these beverages at social and special events. These findings suggest that messages focusing on short-term health consequences may be protective.

This Research Topic also yielded innovative approaches for understanding the high prevalence of child overweight and obesity. Reporting from the Arab countries, Habib-Mourd et al. describe a novel public–private partnership to advance healthy nutrition and physical activity among school-age children. Their framework offers a potentially sustainable, culturally tailored model that could be delivered at scale for primary prevention. Karssen et al. report on an app-based program to promote healthy parenting practices early in life. Promising growth outcomes were observed after 6 months among children with parents randomized to the app condition compared to children in the waitlist-control group. Nezami et al. also report on an mHealth intervention that engaged adults with overweight and obesity and their child. At the 6-month follow-up, they observed an inverse dose-response relationship between parent app use and the proportion of calories from fat and the overall collateral benefits between parent dietary changes and child intake.

Multiple papers investigated strategies that help to understand the association between parenting practices, home and family environment, and child factors. Papaioannou et al. advance science about parent feeding styles, dietary quality, and weight in Hispanic families with low household incomes. While most research has been cross-sectional, they report findings about the directionality of the influence with a prospective longitudinal study. Specifically, an authoritarian feeding style may offer protection in the self-regulatory processes around child appetitive traits. Eagleton et al. also report findings from a prospective longitudinal study in early life. They identify pressure-based feeding and the use of food to soothe as intervention targets to protect against infant food responsiveness. In another prospective study, Loth et al. report on the use of an ecological momentary assessment to evaluate practices among parents of preschool-aged children. Their findings underscore the need to understand how context influences parenting practices and the need to better support parents in response to stress and other factors. Finally, Larsen et al. conduct a systematic review related to parenting practices with a focused lens on families in a lower socioeconomic position. The structural and social factors that parents face warrant attention to promote healthy growth and development. Specifically, targeting structure-related food parenting practices that make healthy foods available and accessible should be regarded as a high priority to establish a more protective environment.

This Research Topic identifies research gaps, informs on the translation of evidence into practice, and may inform future policy guidance to better advance public health objectives related to healthy childhood weight.

## Author contributions

LB-D and JS equally contributed to conceptualizing the Research Topic, reviewing manuscripts, and summarizing the collective work as an Editorial. All authors contributed to the article and approved the submitted version.

## References

[1] U.S. Department of Agriculture and U.S. Department of Health and Human Services. Dietary Guidelines for Americans, 2020-2025, 9th ed. (2020). Available online at: http://DietaryGuidelines.gov (accessed March 2, 2023).

